# Supplementary material for: Discovery of coexisting Dirac and triply degenerate magnons in a three-dimensional antiferromagnet
Source: Nat Commun. 2018 Jul 3;9:2591. doi: 10.1038/s41467-018-05054-2 (PMC6030121; doi:10.1038/s41467-018-05054-2)
Supplement: Supplementary file 1 — Supplementary Information [file 41467_2018_5054_MOESM1_ESM.pdf]

Supplementary Information for
Discovery of coexisting Dirac and triply degenerate magnons in a three-dimensional antiferromagnet

Bao *et al.*

Supplementary Note 1: Effective Hamiltonian of triply degenerate points

Due to the PT symmetry, all magnon bands in Cu_3TeO_6 have twofold degeneracy. The magnonic Hamiltonian can be written as

$$H = H_1 \oplus H_1^*. \tag{1}$$

Near the triply-degenerate nodal point, the linearized $\boldsymbol{k} \cdot \boldsymbol{p}$ Hamiltonian takes the form

$$H_1(\boldsymbol{q}) = E_0 \boldsymbol{I}_{3\times 3} + \sum_{\alpha=x,y,z} v_\alpha q_\alpha S_\alpha, \tag{2}$$

where $\boldsymbol{q} = \boldsymbol{k} - \boldsymbol{k}_0$ is the momentum deviation from the crossing point at $\boldsymbol{k}_0$, E_0 is the energy of the nodal point, v_α is the group velocity, and S_α are the 3×3 matrix representation of the spin-1 operators. Equation 2 gives an effective description of the magnonic dispersions around the H point shown in Supplementary Figure 6.

Supplementary Figures

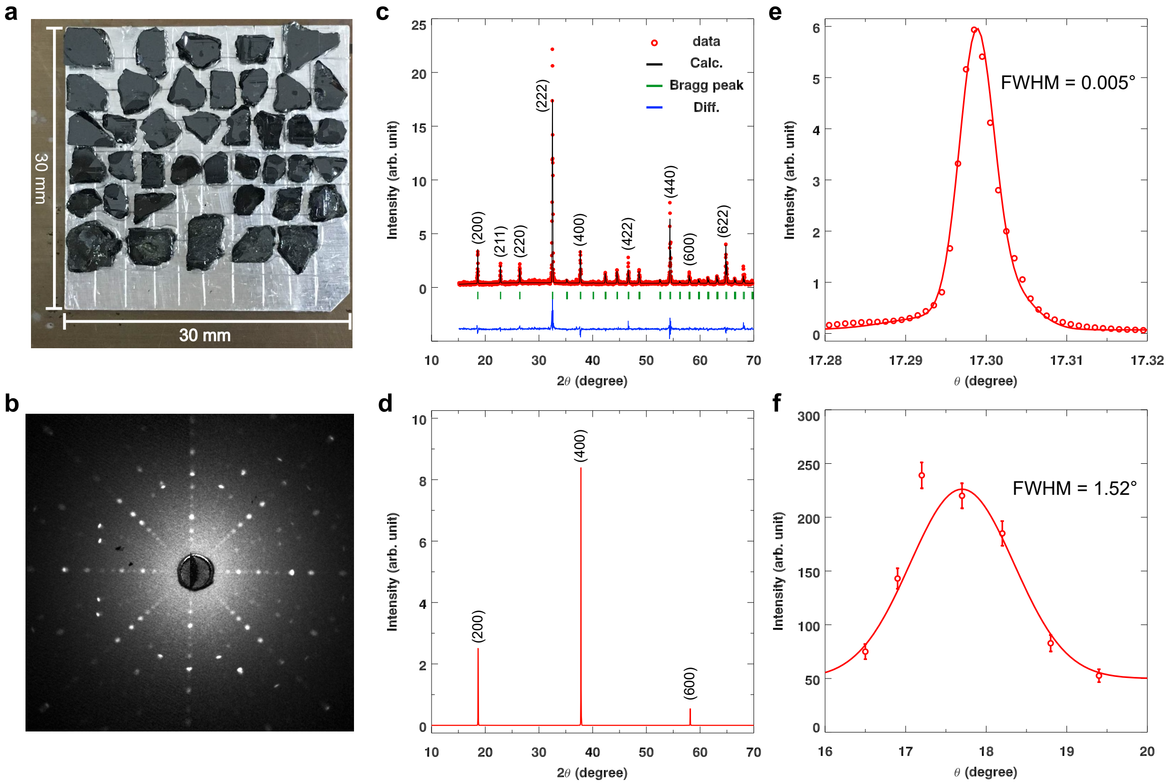

Supplementary Figure 1. Single crystals and structural characterisations. **a**, A photograph of the 40 coaligned single crystals of Cu_3TeO_6 with a total mass about 3 g on an aluminum plate. The natural cleavage plane is the (100) plane. **b**, Laue X-ray pattern of a single crystal with the beam along the [100] direction. **c**, Powder X-ray diffraction pattern with indices for major Bragg peaks. **d**, Single-crystal X-ray diffraction pattern for the (100) plane. **e** and **f**, Rocking curves of single crystals for the (200) peak measured with X-ray and neutrons, respectively. FWHM represents full width at half maximum. Errors represent one standard deviation throughout the paper.

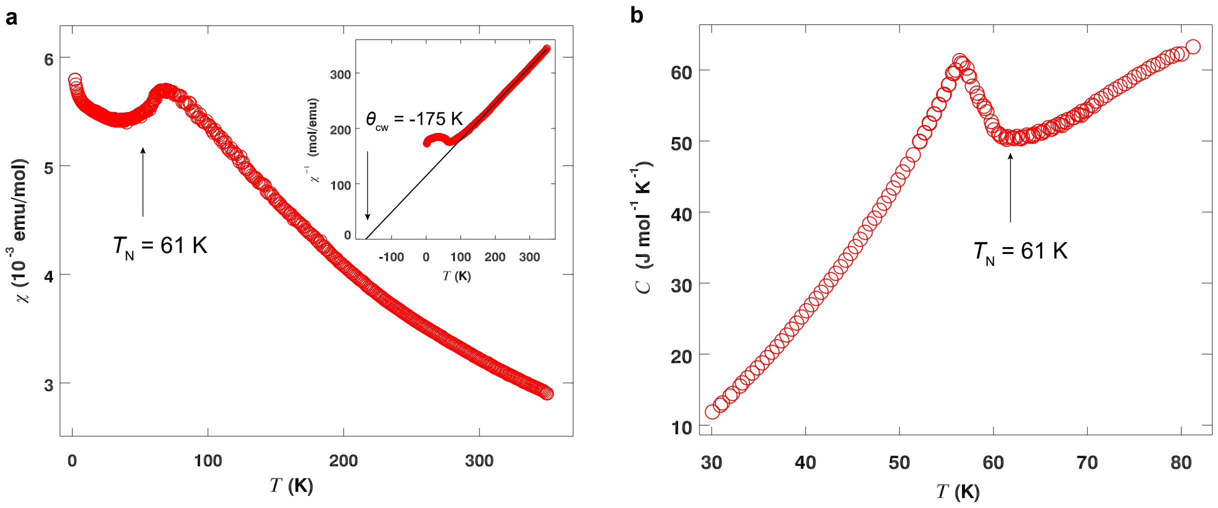

Supplementary Figure 2. Magnetic susceptibility and specific heat. **a**, Magnetic susceptibility of a single crystal measured with a magnetic field of 1 T applied along the [100] direction. The inset shows the inverse susceptibility with a Curie-Weiss fit for temperatures ranging from 150 to 350 K, indicated by the solid line. Θ_{CW} is the Curie-Weiss temperature resulting from the fit. **b**, Specific heat as a function of temperature. The magnetic transition temperature $T_{\mathrm{N}} \sim 61$ K is indicated in both **a** and **b**.

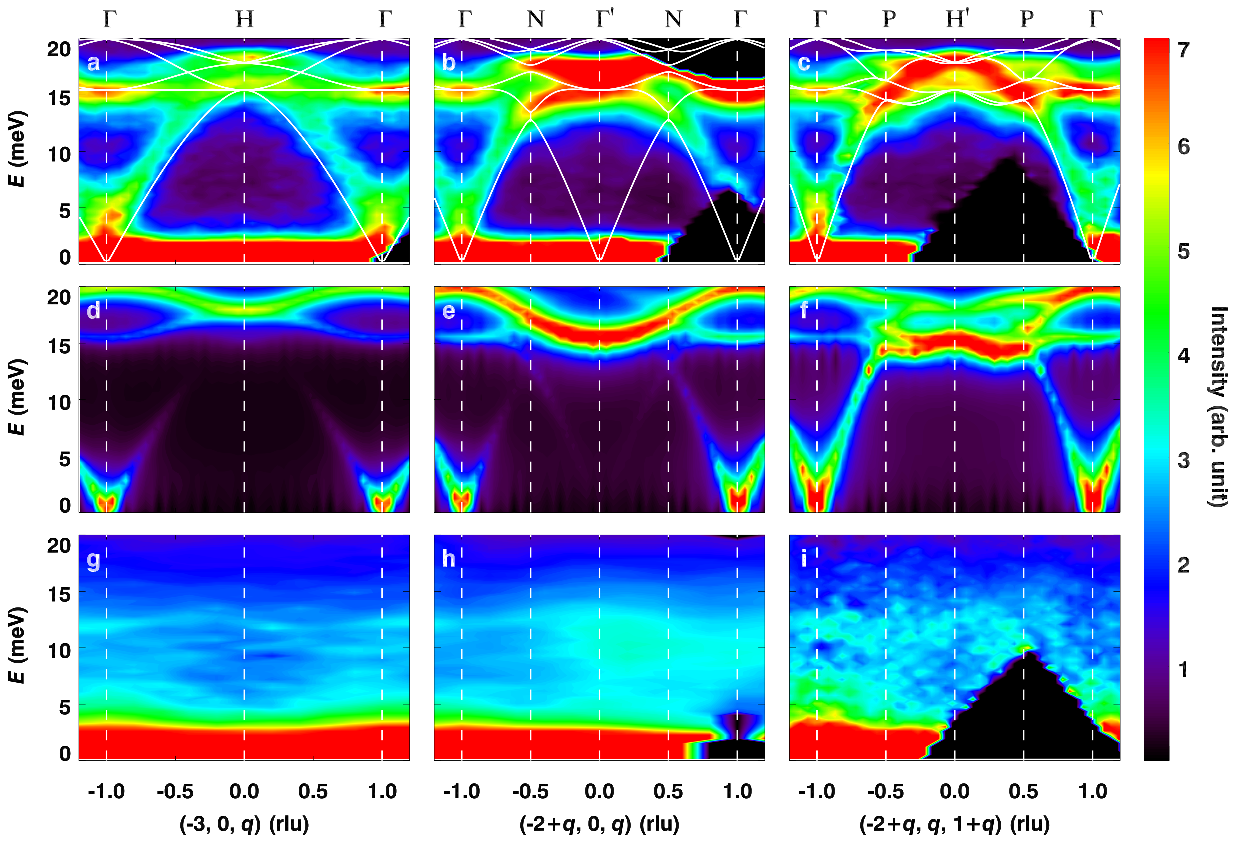

Supplementary Figure 3. Experimental and calculated spin-wave excitations. **a-c**, Inelastic neutron scattering results of the spin excitation spectra measured at $T = 5$ K along [001] (**a**), [101] (**b**), and [111] (**c**) directions, respectively. **d-f**, Calculated magnetic spectra using the linear-spin-wave theory based on a Hamiltonian with nearest-neighbour (NN) exchange interaction J_1 and next NN exchange interaction J_2. The calculated dispersions are plotted as solid lines in **a-c**. Here, J_1 and J_2 are 10.99 and 1.01 meV, respectively. Vertical dashed lines indicate the **Q** (wave vector) positions illustrated in Fig. 1**b** in the main manuscript. **g-i**, Same as in **a-c** but measured at $T = 70$ K.

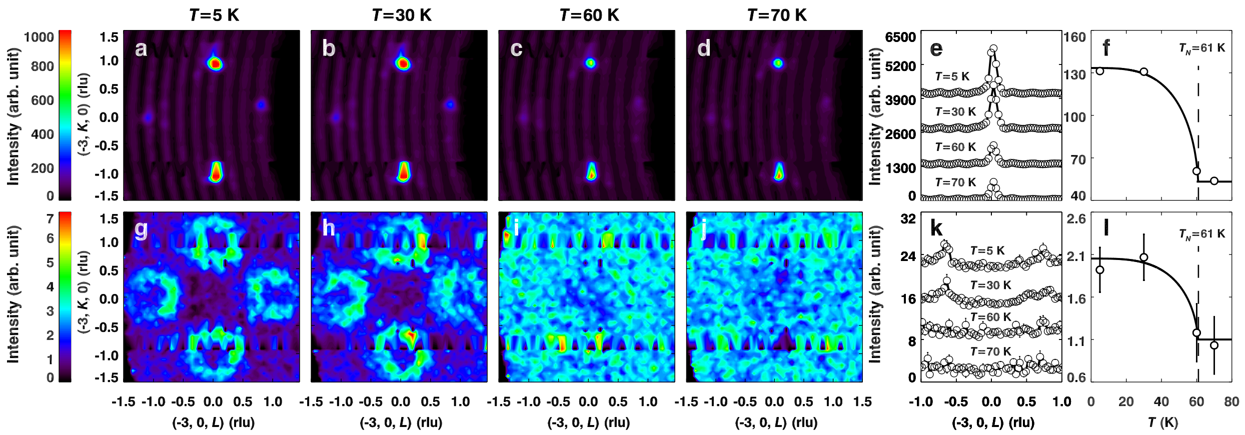

Supplementary Figure 4. Temperature dependence of the magnetic Bragg peaks and spin-wave excitations. **a**, **b**, **c**, and **d**, Constant-energy cuts integrated over the energy range [-2,2] meV at 5, 30, 60, and 70 K, respectively. **g-j**, Similar as in **a-d** but with energies integrated over [8,10] meV. **e**, Constant-**Q** cuts along the [001] direction through $K = [0.9, 1.1]$ rlu in **a-d**. **k**, Similar as in **e** but through $K = [-0.1, 0.1]$ rlu in **g-j**. The intensities of the cuts in **e** and **k** are offset for a better visualisation. **f** and **l**, Integrated intensities over $L = [-1, 1]$ rlu in **e** and **k**, respectively. Solid curves in **f** and **l** are guides to the eye. Dashed lines denote the magnetic transition temperature $T_{\rm N} \sim 61$ K. Data in **a-d** and **g-j** are integrated over $[H, 0, 0] = [-3.2, -2.8]$ rlu. Intensities of elastic scattering at $T = 70$ K in **d-f** are due to nuclear scattering, which remain finite above $T_{\rm N}$.

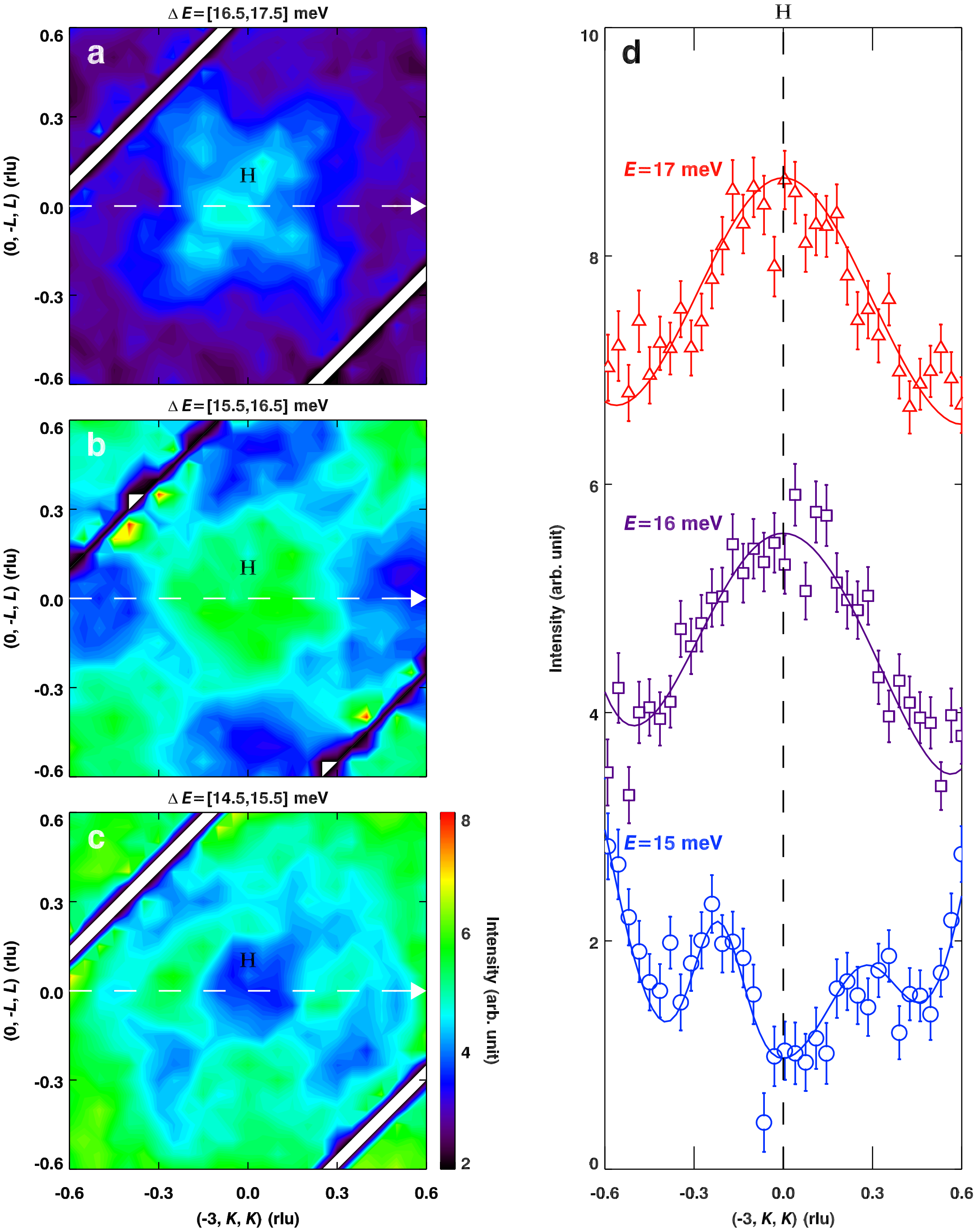

Supplementary Figure 5. A triply degenerate node at a lower energy of 16 meV. **a-c**, Contours plotted against two orthogonal axes [$\bar{3}$11] and [0$\bar{1}$1] with different energy intervals. Dashed arrows indicate the trajectory of the cuts plotted in **d**. The vertical dashed line in **d** denotes the position of the triply degenerate node. Lines through data are fits with Gaussian functions. Intensities in **d** are offset according to the energy. These results illustrate that the bands cross each other at the H point.

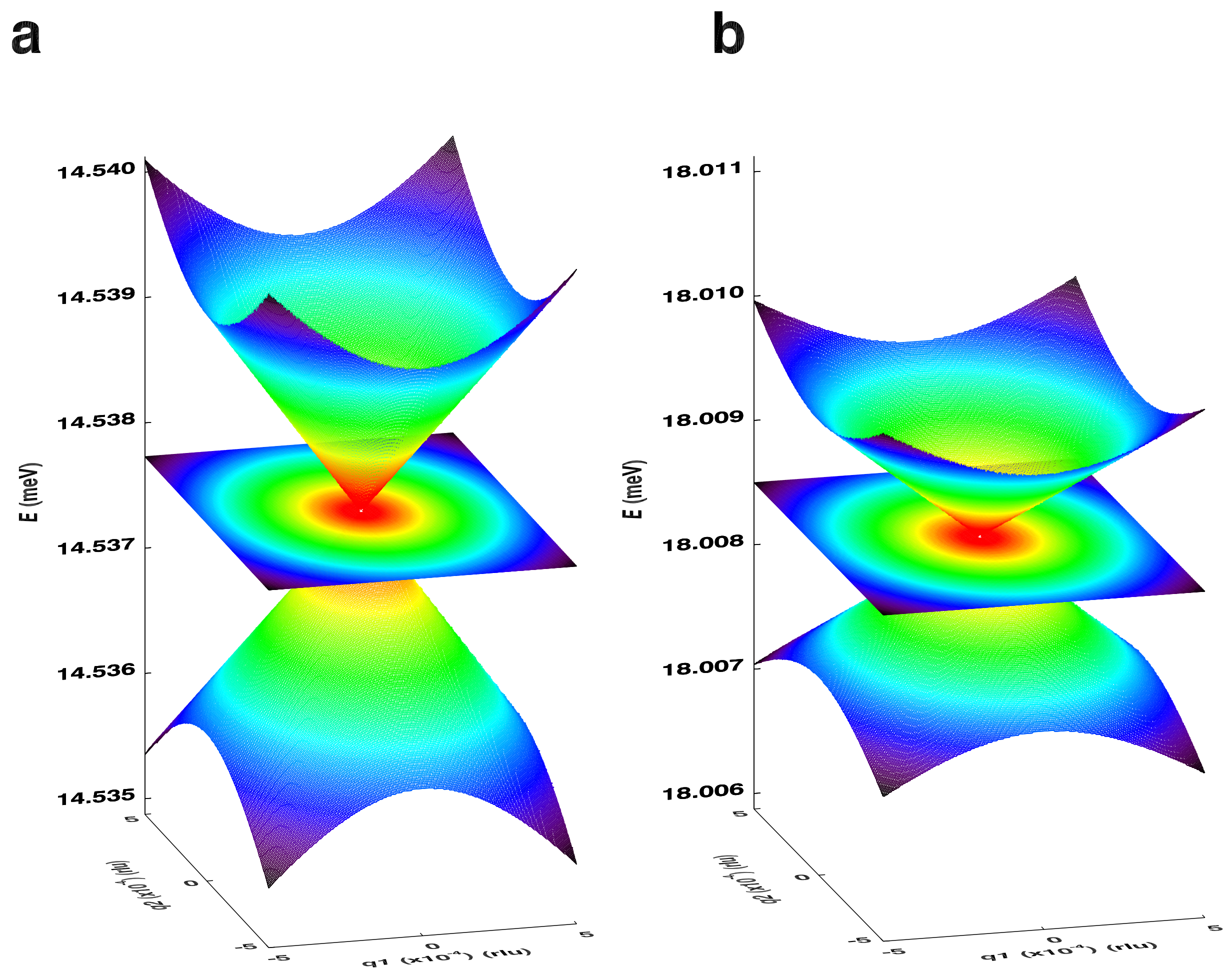

Supplementary Figure 6. Calculated dispersions near triply degenerate points. Calculated dispersions near the H point based on Eq. 1 in the main manuscript. Three magnon bands cross at the H point at two different energies. The magnon bands can be described by Supplementary Equation 2 effectively.
